# Serial transfer can aid the evolution of autocatalytic sets

**DOI:** 10.1186/1759-2208-5-4

**Published:** 2014-04-26

**Authors:** Wim Hordijk, Nilesh Vaidya, Niles Lehman

**Affiliations:** SmartAnalytiX.com, Lausanne, Switzerland; Department of Chemistry, Portland State University, PO Box 751, Portland, OR 97207 USA; Department of Chemical and Biological Engineering, Princeton University, Princeton, NJ 08544 USA

**Keywords:** Serial transfer, RNA, Gillespie algorithm, Origins of life, Autocatalytic set

## Abstract

**Background:**

The concept of an autocatalytic set of molecules has been posited theoretically and demonstrated empirically with catalytic RNA molecules. For this concept to have significance in a realistic origins-of-life scenario, it will be important to demonstrate the evolvability of such sets. Here, we employ a Gillespie algorithm to improve and expand on previous simulations of an empirical system of self-assembling RNA fragments that has the ability to spontaneously form autocatalytic networks. We specifically examine the role of serial transfer as a plausible means to allow time-dependent changes in set composition, and compare the results to equilibrium, or “batch” scenarios.

**Results:**

We show that the simulation model produces results that are in close agreement with the original experimental observations in terms of generating varying autocatalytic (sub)sets over time. Furthermore, the model results indicate that in a “batch” scenario the equilibrium distribution is largely determined by competition for resources and stochastic fluctuations. However, with serial transfer the system is prevented from reaching such an equilibrium state, and the dynamics are mostly determined by differences in reaction rates. This is a consistent pattern that can be repeated, or made stronger or weaker by varying the reaction rates or the duration of the transfer steps. Increasing the number of molecules in the simulation actually strengthens the potential for selection.

**Conclusions:**

These simulations provide a more realistic emulation of wet lab conditions using self-assembling catalytic RNAs that form interaction networks. In doing so, they highlight the potential evolutionary advantage to a prebiotic scenario that involves cyclic dehydration/rehydration events. We posit that such cyclicity is a plausible means to promote evolution in primordial autocatalytic sets, which could later lead to the establishment of individual-based biology.

## Background

### Autocatalytic sets

An autocatalytic set is a collection of molecular species in which the network as a whole forms a functionally closed and self-sustaining system [1]. This network-based perspective, as opposed to an individual-based one, is becoming recognized as a plausible early stage in the emergence of life. In the past decade, this idea has been explored formally, by casting it into graph and probability theory [2, 3] and examining its underlying chemical dynamics [4, 5], by generalizing it to other systems [4, 6], and by exploring the features of minimal sets that fit its definition [7]. In fact the relevance of autocatalytic sets to a wide variety of phenomena is now becoming apparent [8].

Only very recently though, has progress in the laboratory in creating actual such sets with informational molecules [7–13] enabled a powerful convergence of empirical and theoretical approaches to their study. These works were inspired by earlier studies with short nucleic acid oligomers [14]. RNA, through specific nucleotide-pairing interactions that allow certain sequences to recognize each other and discriminate among similar sequences, has the ability to spontaneously form complex networks. Moreover, if these RNAs are catalytic in that they can augment the rate of synthesis of other RNAs, an autocatalytic network can be set up [10]. Previous numerical simulations of such networks have emulated well the interaction events observed in the laboratory, and furthermore confirm that cooperativity in such networks can lend RNA with a competitive advantage over selfish systems that are less able or unable to form autocatalytic sets [15]. Yet the creation of true autocatalytic sets in the laboratory is still a challenging process, making it therefore essential to have more realistic and accurate computer models to make progress in this field.

Our work before has exploited the fact that the *Azoarcus* ribozyme, which is roughly 200 nucleotides (nt), can be broken into up to four shorter oligonucleotide fragments (termed **W**, **X**, **Y**, and **Z**) and that these fragments can spontaneously reassemble into covalent versions of the ribozyme. The chemistry employed for this is *trans*-esterification, which effectively means that RNA fragments can be catalytically recombined into new, larger RNAs. The assembly can happen from four pieces:1

but one can also set up the reaction to occur from various combinations of only two pieces:234

In any of these cases, the dot (•) represents a covalent bond and the reactions are catalyzed either by non-covalent assemblages of ribozymes that form through base-paring and 3˚ interactions or by partially or fully covalent versions of the ribozyme through autocatalytic feedback. Importantly, the specificity of these reactions is guided through specific nucleotide triplet-triplet interactions (see “The RNA system”, below). These triplets can be altered in a way that rather complicated networks of RNAs can be created in which covalent assembly requires spontaneous cooperation between other sets of RNAs and the compositions of these networks can be observed to change in real time [10]. We have previously modeled some aspects of this system and found that many of its parts can be parameterized and that in general there is good correspondence between expected and observed behavior, particularly in regard to RNA-RNA cooperation dynamics [15].

An immediate requirement of both empirical and simulation studies of RNA autocatalytic sets is that they be able to accommodate evolutionary principles. These sets must be able to change their composite species frequencies over time and respond to selection pressures if they are to be relevant models of historical events on the prebiotic Earth. In principle, autocatalytic sets can evolve by expanding (including more members), contracting (expelling some members), and/or by changing the identities and relative concentrations of their composite members. Note that these evolutionary events are non-Darwinian in the traditional sense because the relative fitnesses of individual genotypes is not the underlying driving force for persistence. Nevertheless the frequency changes of network members – compositional changes – with time, should they be based on selectable forces, would represent a primitive sort of evolutionary change that has closer ties to chemistry rather than biology.

These sorts of evolutionary processes are difficult to envisage in a static, or “batch”, environment once the system reaches equilibrium. Thus there is a need to explore realistic models of environmental change that permit network evolution. A serial drying and rehydration of an aqueous environment is a commonly invoked feature of the prebiotic Earth [16–18] and one in which many types of prebiotic chemistry – including those associated with nucleic-acid synthesis – actually become facilitated. Here, we explore the efficacy of a common laboratory mimic of one potential consequence of dehydration/rehydration cycles, namely serial dilution, to promote the evolution of autocatalytic sets. We do this by expanding and improving the match between a previous model [15] and the chemical details of an RNA autocatalytic set [10], and simultaneously investigate the effects of serially transferring a fraction of one mixture of RNA molecules into a naïve environment containing new food-stock for RNA synthesis.

### The RNA system

We based our simulations on an RNA system in which composite RNA oligonucleotides, ranging in size from ~40–150 nt, can spontaneously self-assemble into a recombinase ribozyme [19]. These are fragments of the *Azoarcus* self-splicing group I intron [20], which when fully assembled through contiguous covalent bonds, is roughly 200 nt in length. This ribozyme can catalyze further covalent synthesis of other such ribozymes, creating an autocatalytic feedback loop [21]. The initiation of this catalytic cycle is made possible by the non-covalent association of RNA fragments (here, “food” molecules) into loose coalitions that possess a small amount of activity towards recombining other fragments into covalently joined molecules [19].

Information transfer in this chemical system is mediated by a 3-nt base-pairing interaction between the internal guide sequence (IGS) located on the 5´ end of the ribozyme, and corresponding pseudo-complementary 3-nt “tag” sequences located on the 3´ ends of each RNA fragment. In other words, catalysts (either the loose coalitions or the covalently contiguous forms of the ribozyme), can see and discriminate among other RNAs on the basis of this 3-nt interaction. To a first approximation, an IGS triplet that does not make strong Watson-Crick base pairs with a tag will not promote assembly using the RNA that contains that tag. Thus, interactions can be described as either “cooperative” (if an IGS base pairs with tags on other molecules with different IGS sequences) or “selfish” (if an IGS only base pairs with tags on molecules with the same IGS sequence). With this discriminatory situation, various sorts of interaction networks can be established [10]. To examine what sorts of ribozymes might emerge from a simulated pre-biotic scenario, we previously mixed fragments of these RNAs in which the middle nucleotide of the IGS (symbolized here as M) and that of the tags (symbolized here as N) were randomized. We were able to observe the synthesis of various frequencies of covalently contiguous full-length (200 nt) ribozymes over time in both batch (*i.e*., one-pot) scenarios and serial transfer scenarios in which 10% of the RNA solution was transferred to a new tube every hour [10]. For the batch scenario, the establishment of equilibrium after a few hours of reaction time revealed that cooperativity could in some cases produce higher concentrations of full-length ribozymes than selfishness, and this was corroborated and explained using ODE and stochastic simulation models [10, 15]. However the serial transfer case was not analyzed, and the full stochastic nature of the system requires enhancements to the model. Here we explore both of these aspects.

## Experimental

### The model

We have constructed a formal model of the experimental RNA system and used the Gillespie algorithm to simulate its dynamical behavior. An earlier, but simpler, model was described previously [15]. Here we include more chemical realism in the model and perform a more detailed analysis. To fully specify the model, we first need to define the molecular species, the possible chemical reactions, which molecules catalyze which reactions, and the corresponding reaction rates. Then the simulation algorithm is described in detail.

#### Molecules

There are three main groups of molecule types. First, there are the RNA fragments (first group). These constitute the “food set” in the context of autocatalytic sets. Second, these RNA fragments associate spontaneously into non-covalent ribozymes (second group). And third, the non-covalent ribozymes are transformed (through catalyzed reactions) into the corresponding covalent ribozymes (third group).

For the purposes of the model, in the first group of molecule types (the RNA fragments), we ignore the specific junction at which the RNA fragments are covalently recombined, and only consider the particular combination of nucleotides in the IGS and tag sequences of the resulting (non-covalent) ribozyme to be important. Since the RNA fragments are oriented (left to right, or 5´ to 3´), we assume there is a “left” (l) fragment and a “right” (r) fragment that associate, each of which can have any of the four possible nucleotides in the IGS/tag sequence. Consequently, there are four possible “left” fragments and four possible “right” fragments, *i.e*., eight RNA fragments constituting the first group of molecule types (the food set). We label these l_M_ and r_N_, M,N∈{A,C,G,U}.

Given an association of such a “left” fragment and a “right” fragment, there can be 16 possible resulting non-covalent ribozymes (second group of molecule types), labeled I_MN_, M,N∈{A,C,G,U}, depending on the relevant nucleotides M and N that are combined.

Finally, there are the 16 corresponding covalent ribozymes (third group of molecule types), labeled E_MN_, M,N∈{A,C,G,U}. Thus, in total there are 8 (first group) + 16 (second group) + 16 (third group) = 40 molecule types in the model.

#### Reactions

There are two main groups of reactions. First, there are the (spontaneous) association reactions transforming two RNA fragments into a non-covalent ribozyme: l_M_ + r_N_ → I_MN_. There are 16 such reactions (one for each of the 16 possible non-covalent ribozymes). We also include the reverse (dissociation) reactions in the model.

Second, there are the catalyzed recombination reactions that convert a non-covalent ribozyme into a covalent one: I_MN_ → E_MN_. There are 16 such reactions (one for each of the 16 possible ribozymes), and they can be catalyzed by either a non-covalent or a covalent ribozyme (see below). We also include the reverse reactions in the model. Thus, in total there are 16 + 16 = 32 (bi-directional) reactions in the model.

#### Catalysis

The ribozymes (both non-covalent and covalent) catalyze each others’ transformation from non-covalent to covalent. In particular, if a ribozyme E_MN_ (or I_MN_) has a nucleotide M in its guide sequence that is the base-pair complement of the variable nucleotide N' in the tag sequence of another ribozyme I_M'N'_, then the first ribozyme E_MN_ (I_MN_) can catalyze the non-covalent to covalent transformation of the second ribozyme I_M'N'_. So, each ribozyme catalyzes the transformation reaction of four other ribozymes (or possibly of three others and that of itself). There is a difference in rates depending on whether the catalyst is a non-covalent (I_MN_) or covalent (E_MN_) ribozyme (see below).

The RNA fragment association reactions (forming non-covalent ribozymes) are spontaneous. In the formal autocatalytic sets framework, spontaneous (non-catalyzed) reactions can by definition never be part of an autocatalytic set. This is because, usually, spontaneous reactions happen at significantly lower rates than catalyzed ones, often too low to be relevant. However, in the RNA system the spontaneous RNA-RNA association reactions actually happen at very high rates because these aggregations are driven by favorable base-paring and tertiary interactions, which are numerous in the *Azoarcus* ribozyme [22]. In the formal framework, we can incorporate such spontaneous but high-rate reactions by assuming that they are actually catalyzed by a (fictional) “generic catalyst” which is also part of the food set. Here, this assumption is made implicitly, but not explicitly included in the model.

#### Rates

Relative rates for the transformation reactions were obtained experimentally by steady-state kinetic analyses of representative matching and non-matching (*i.e*., Watson-Crick) IGS-tag relationships in RNA fragments [10, 21]. These rates were then re-scaled to make one time unit in the simulation correspond roughly to one hour in the real experiments. In these experiments, at the end of each transfer step (one hour), close to 20% of the solution has been transformed into covalent ribozymes [10]. Using this as a target, the experimentally obtained rates were then rescaled (while maintaining their relative ratios) such that after one time unit in the simulation also close to 20% of the solution consists of covalent (E_MN_) ribozymes.

The transformation reaction rates depend on the particular base-pair combination of the relevant nucleotide in the IGS of the catalyst and that of the tag sequence in the reactant. The following (re-scaled) rates were obtained for the four possible Watson-Crick base-pair combinations (ordered from high to low rates):

AU: 0.00613, CG: 0.00541, UA: 0.00517, and GC: 0.00445.

For example, the transformation reaction I_CU_ → E_CU_ catalyzed by the covalent ribozyme E_AG_ has a rate of 0.00613 (catalyst/reactant base-pair combination AU).

The rates of non-covalent ribozyme catalyzed transformation reactions are set to half the rates of the corresponding covalent ribozyme catalyzed reactions, as observed in the laboratory experiments [10]. The rates of the spontaneous association reactions in the simulation model are set to 0.00006 (same rate for each of the 16 possible association reactions). Finally, the rates for all reverse reactions were set to 1/10th the rate of their corresponding forward reactions.

#### Simulation

To simulate the dynamics of the above chemical reaction system, we used the Gillespie algorithm [23, 24] assuming a closed reaction vessel (with volume 1). Starting with an initial amount of 2000 of each of the 8 food molecule types (*i.e*., a total amount of 16,000 molecules), the algorithm is run for a given number of time units *t*. We performed two types of simulations: (*i*) with and (*ii*) without transfer steps.

In a simulation without transfer steps, the Gillespie algorithm is simply run for the given number of time units. In a simulation with transfer steps, at the end of each time unit first the solution is diluted to 10%. Rather than simply reducing the concentration of each molecule type to 10%, this dilution is done by randomly drawing molecules (without replacement) from the solution with probabilities according to their relative current concentrations, until 10% of the total concentration is reached. Next, from this diluted solution a random sample (with replacement) of 75 molecules is taken from among the 16 E_MN_ types. Each E_MN_ type that occurs twice or more in this random sample is reported. Finally, the diluted solution is replenished with the 8 food molecule types (in equal concentrations) until the initial total concentration of 16,000 is reached again. These transfer steps are then repeated for the given number of time units. This provides a detailed simulation of the original laboratory serial transfer experiments [10], which can be repeated an arbitrary number of times.

As a consequence of computational limitations, the total amount of molecules used (16,000) is relatively small, and clearly does not compare to the micromolar concentrations in the real experiments. However, to get an idea of how the system’s behavior scales with larger numbers of molecules, we also performed a number of runs of the same simulation but with two orders of magnitude more food molecules (starting with 200,000 molecules of each food type, or a total of 1.6E6 molecules, and appropriately adjusting the time units to maintain a close to 20% conversion to E_MN_ ribozymes after one time unit). Even though this is still a small number compared to the laboratory experiments, we argue that in an origin-of-life context the actual concentrations were most likely significantly lower than micromolar, giving more relevance to the results presented here.

## Results and discussion

**Figure 1:**
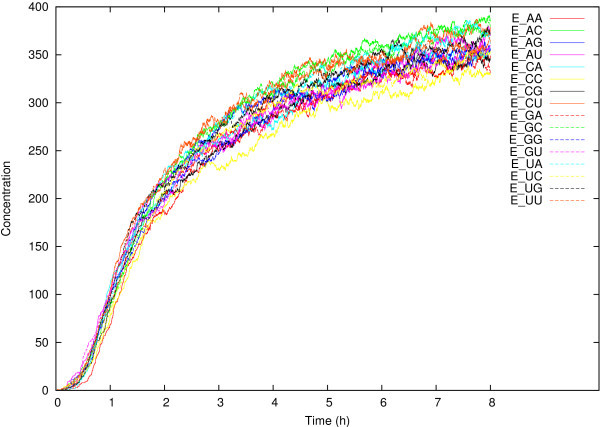
**The concentrations of the covalent ribozymes E**_**MN**_ **over time in a typical run of the simulation model (starting with 16,000 food molecules) without transfer steps, for a total of** ***t*** **= 8 time units.**

We first ran the simulation model without transfer steps for *t* = 8 time units. Figure 1 shows the concentrations of the 16 covalent ribozymes E_MN_ over time for one such run. By the end of the run, the system seems to have reached an equilibrium state. Given an initial concentration of 2000 for each of the food molecule types (RNA fragments), each E_MN_ type can potentially reach a total concentration of 500. However, due to the stochastic nature of the Gillespie algorithm (as in real chemistry), the RNA fragments will not associate into the intermediate non-covalent ribozymes in exactly equal amounts. There is competition among the different E_MN_ molecules for the same resources, and the relative concentrations in the equilibrium state are thus largely a result of stochastic fluctuations in the production of the different non-covalent ribozymes. For example, in one typical run of the simulation, the total concentrations of I_MN_ + E_MN_ molecules for the 16 different types at the end of the run (eight time units) vary from 390 to 431, a difference of 41, or around 10%.

Furthermore, due to the reverse reactions [25], reflected in the wiggly lines in Figure 1, the equilibrium state is a dynamic one. Covalent ribozymes can be transformed back into non-covalent ones, which can dissociate again into RNA fragments (at a rate of 1/10^th^ of the corresponding forward reactions). As a consequence, the potential concentration of 500 is not reached and, moreover, the relative concentrations still change over time, even in the equilibrium state. Indeed, ordering the 16 E_MN_ molecule types according to their final concentrations (at *t* = 8) gives a different (and seemingly random) order in different simulation runs.

**Figure 2:**
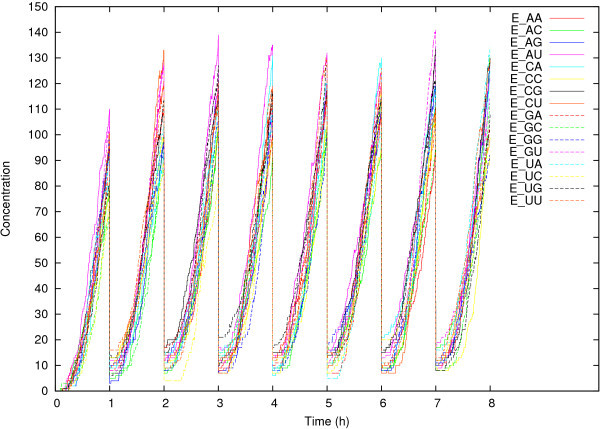
**The concentrations of the covalent ribozymes E**_**MN**_ **over time in a typical run of the simulation model (starting with 16,000 food molecules) with transfer steps, for a total of** ***t*** **= 8 time units.**

Next, we ran the simulation model with transfer steps, also for *t* = 8 time units (as in the original chemical experiments). Figure 2 shows the concentrations of the 16 covalent ribozymes E_MN_ over time for one such run. The effect of the transfer steps can clearly be seen. At the end of each time unit, the concentrations are roughly around 100 (*i.e*., 20% of the potential 500), which are diluted to about 10 during the transfer step. These concentrations then quickly start increasing again due to the resupply of food molecules at the end of each transfer step.

**Figure 3:**
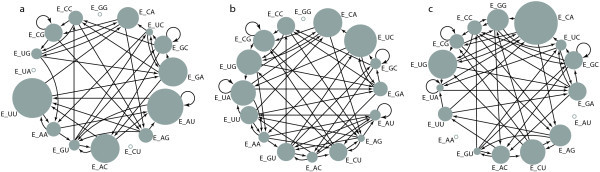
**The varying autocatalytic (sub)sets as observed during one particular simulation run at the end of transfer steps number 1 (a), 3 (b), and 8 (c).** The sizes of the circles indicate the relative frequencies of the different E_MN_ molecules in the sample of size 75 taken after each dilution. Empty circles indicate genotypes that do not occur at least twice in the sample, as in the original experiment [10]. Arrows indicate the catalytic relationships, but the frequency changes depicted in Table 1 are the result of network dynamics, not merely single inter-genotype interactions.

These simulation results seem to reproduce the experimental results quite well. For example, Figure 3 shows the results of taking a sample of size 75 from among the E_MN_ molecules (after the dilution to 10%) at different time steps. These graphs are indeed very similar to the experimental ones (Figure four in ref [10]). Also, they confirm the previous model prediction [15] that a different sequence of networks is observed each time the experiment/simulation is repeated, due to the stochastic nature of the system.

The transfer steps clearly prevent the system from reaching an equilibrium state. It turns out that this has an important impact on the relative concentrations of the covalent ribozymes. Whereas these relative concentrations in the equilibrium state are largely a consequence of competition for resources (as explained above), on short time scales, when there are still abundant food molecules available, they are largely determined by differences in the reaction rates.

This distinction is shown in Table 1. The first column shows the average concentrations of the covalent ribozymes (starting with 16,000 food molecules and averaged over 50 simulation runs) after one time unit (*t* = 1), and ordered from highest to lowest. The second column shows the same but after eight time units (*t* = 8; no transfer steps performed). Clearly, after only one time unit, the covalent ribozymes that have the highest rate of production (the E_*U_ molecules with rate 0.00613) also have the highest concentrations on average (first four molecule types in the first column). Furthermore, the ribozymes with the lowest rate of production (the E_*C_ molecules with rate 0.00445) have the lowest average concentration (last four molecule types in the first column). The other two molecule types (E_*A_ and E_*G_), which have similar rates in between (0.00517 and 0.00541, respectively), are mixed and between the E_*U_ and E_*C_ molecules. This pattern is the same on any set of 50 runs over which the averages are calculated (although the order of the four E_*U_ molecule types within the top four might be different, and similarly for the order of the four E_*C_ molecule types within the bottom four).

**Table 1:** **Short-term vs long-term dynamics**

16,000 food molecules				1,600,000 food molecules			
***t***= 1		***t***= 8		***t***= 1		***t***= 8	
UU	101.1	CA	365.0	CU	9899	CG	36200
AU	100.8	AG	364.8	UU	9888	UA	36160
CU	99.0	GU	363.4	GU	9875	AU	36145
GU	98.5	GG	363.3	AU	9873	GU	36139
AG	96.3	UU	362.1	AG	9290	GG	36121
CA	94.3	AC	361.4	GG	9276	CU	36116
UG	92.5	AA	360.3	UG	9272	UG	36112
GA	91.8	GC	360.2	CG	9268	AA	36094
GG	91.5	CG	360.1	CA	9054	AC	36092
AA	90.3	UG	359.8	AA	9043	GA	36083
UA	90.1	CC	359.6	GA	9022	CA	36082
CG	90.0	AU	359.6	UA	9017	UC	36072
AC	83.9	UC	359.2	GC	8305	AG	36069
GC	83.7	UA	358.7	CC	8302	CC	36059
CC	82.5	GA	358.6	UC	8300	GC	36054
UC	81.6	CU	357.1	AC	8257	UU	36053

The average number of molecules of the 16 covalent ribozymes after one time unit (*t*=1) and eight time units (*t*=8) without transfer steps, ordered from high to low. The left half of the table is for simulations starting with 16,000 food molecules (2,000 of each type), averaged over 50 runs. The right half of the table is for simulations starting with 1,600,000 food molecules (200,000 of each type), averaged over 20 runs.

However, after eight time units the situation is very different, as already observed earlier. There is no clear pattern in the ordering (second column) and, in fact, the ordering is completely different (and seemingly random) over different sets of 50 runs. Furthermore, the variance in the average concentrations is much lower. After one time unit, the difference between the highest and lowest concentrations is 101.1 – 81.6 = 19.5 (which is 19.3% of the highest concentration). However, after eight time units, this difference is 365.0 – 357.1 = 7.9 (which is only 2.2% of the highest concentration). So, not only in absolute value, but also in relative value the variance in concentrations is much higher after only one time unit than after eight time units.

This pattern of the relative concentrations being largely determined by the differences in reaction rates is still clearly visible after two time units (no transfer steps) as well, although with a lower variance in concentrations (9.84, or 4.6% of the highest concentration). However, after four time units (no transfer steps), the pattern has already disappeared, and the ordering is more or less random and the variance very low, just as after eight time units. This pattern can be made even stronger by making the reaction rates even more different. For example, increasing the rate of production of E_*U_ molecules and decreasing the rate of production of E_*C_ molecules, the ordered pattern can persist even up to three or four time units (no transfer steps). Obviously if the production rates of all E_MN_ molecules are equal, than the ordering is always random, even already after one time unit.

Now, measuring the relative concentrations of the covalent ribozymes at the end of the simulation run over eight time units and *with* transfer steps, again averaged over 50 runs, shows the same ordered pattern as the first row of Table 1. As mentioned, the transfer steps prevent the system from reaching the (random) equilibrium state and, as a consequence, the relative concentrations are still largely determined by the differences in reaction rates, not by competition for resources. This pattern is the same, no matter how many transfer steps are performed, as long as the duration of the transfer steps is relatively short (on the order of one or two time units). Note that the concentrations at the end of any one particular transfer step do not necessarily reflect this ordered pattern (cf. Figure 3). However, on average (over a larger number of transfer steps), this pattern is clear and consistent.

To see whether this phenomenon is not an artifact of the relatively small number of molecules used in the simulations, we repeated the analysis with a starting concentration of 1,600,000 food molecules (200,000 of each type), and averaging over 20 runs. The results are shown in the third (*t* = 1) and fourth (*t* = 8) columns of Table 1. Clearly, the effect is even stronger in this case, even though the averages are over a smaller number of runs (20 instead of 50, due to higher computational requirements). Now also the two “intermediate” ribozyme types (E_*A_ and E_*G_) are well separated after one time unit. So, we can expect that with even larger concentrations, the effect on selection is even stronger.

## Conclusions

We have improved and expanded our previous model [15] of an autocatalytic set that is observed in a self-assembling RNA system that is a plausible mimic of the origins of information transfer. Our main observation is that serial transfer has the effect of preventing the molecular system from reaching chemical equilibrium, thereby augmenting the power of slight differences among rate constants to promote selection. While a batch system can generate different frequencies of its final products, in the context of network dynamics, the evolutionary potential of such a scenario is highly limited. With serial transfer, we can observe changes in network composition over time (Figure 3) that are dependent and driven by relatively small intrinsic variations in 3-nt base-pairing interactions, whose free energies can differ by less than 1 kcal/mole in the active site of catalytic RNAs such as the ones under study [26]. Our results were obtained even when the composition of the food-stock was kept constant in each transfer; true environmental systems would not be so regular and the effects of serial transfer on selection should be similarly augmented. This phenomenon should be applicable to a wide range of autocatalytic set realizations including protein networks [11], nucleic-acid networks [9, 10], and lipid networks such as the composomes envisioned in the GARD model of Lancet and co-workers [27]. We posit that a pre-biotic scenario of dehydration/rehydration would be permissive for the evolution of autocatalytic networks and thus should be explored further.
